# A Novel Validated Injectable Colistimethate Sodium Analysis Combining Advanced Chemometrics and Design of Experiments

**DOI:** 10.3390/molecules26061546

**Published:** 2021-03-11

**Authors:** Ioanna Dagla, Anthony Tsarbopoulos, Evagelos Gikas

**Affiliations:** 1 Laboratory of Pharmaceutical Analysis, Division of Pharmaceutical Chemistry, Faculty of Pharmacy, School of Health Sciences, National and Kapodistrian University of Athens, Panepistiomiopolis, Zografou, 157 71 Athens, Greece; idagla@pharm.uoa.gr; 2 GAIA Research Center, Bioanalytical Department, The Goulandris Natural History Museum, 14562 Kifissia, Greece; atsarbop@med.uoa.gr; 3 Laboratory of Pharmacology, Department of Descriptive-Functional Studies, Faculty of Medicine, National and Kapodistrian University of Athens, 115 27 Athens, Greece; 4 Laboratory of Analytical Chemistry, School of Chemistry, National and Kapodistrian University of Athens, Panepistiomiopolis, Zografou, 157 71 Athens, Greece

**Keywords:** colistimethate, UPLC–UV, multivariate analysis, pharmaceutical formulations, quality control, partial least square regression

## Abstract

Colistimethate sodium (CMS) is widely administrated for the treatment of life-threatening infections caused by multidrug-resistant Gram-negative bacteria. Until now, the quality control of CMS formulations has been based on microbiological assays. Herein, an ultra-high-performance liquid chromatography coupled to ultraviolet detector methodology was developed for the quantitation of CMS in injectable formulations. The design of experiments was performed for the optimization of the chromatographic parameters. The chromatographic separation was achieved using a Waters Acquity BEH C_8_ column employing gradient elution with a mobile phase consisting of (A) 0.001 M aq. ammonium formate and (B) methanol/acetonitrile 79/21 (*v*/*v*). CMS compounds were detected at 214 nm. In all, 23 univariate linear-regression models were constructed to measure CMS compounds separately, and one partial least-square regression (PLSr) model constructed to assess the total CMS amount in formulations. The method was validated over the range 100–220 μg mL^−1^. The developed methodology was employed to analyze several batches of CMS injectable formulations that were also compared against a reference batch employing a Principal Component Analysis, similarity and distance measures, heatmaps and the structural similarity index. The methodology was based on freely available software in order to be readily available for the pharmaceutical industry.

## 1. Introduction

The increasing resistance of Gram-negative bacteria (GNB) to all known antibiotics (e.g., penicillins, aminoglycosides and β-lactams) and the absence of new, effective drugs against multidrug resistant bacteria (MDR) led to the reconsideration of old-generation antibiotics such as the polymyxins [1,2,3,4]. Colistin (polymyxin E) re-emerged in the 1990s to cover the then-inefficient treatment of MDR–GNB-caused infections [5,6]. It is deemed to be a last-resort in the era of antibiotic resistance; however, it induces high neuro- and nephrotoxicity; thus, its administration is performed carefully to consider the patient’s condition, adjustment of the dosing regimen and assessment of the risk–benefit balance. Colistin can be administered as colistin sulfate (CS) either orally or topically and as colistimethate sodium (CMS) parenterally or by inhalation. CMS is the inactive, less-toxic prodrug of colistin that hydrolyzes in vivo in the active form of colistin [7].

The knowledge of CMS pharmacokinetics is important for determining the dosing regimen to minimize the drug’s toxicity and increase its therapeutic window. Hence, several analytical methodologies have been reported for the indirect determination of CMS in biological fluids after its hydrolysis to colistin, based mainly on liquid chromatography coupled to mass spectrometry (LC–MS) [8,9,10,11,12,13].

CMS is produced by sulfomethylation of colistin’s five free primary amine groups by reductive amination adding formaldehyde and sodium hydrogen sulfite sequentially. Nevertheless, the actual structure of CMS has not been clarified. Barnett et al. have reported that the CMS structure involves the mono-sulfomethylation of one to five amino groups [14], but EMA reported that amino groups are either non-substituted or bis-sulfomethylated [15]. Thus, conflicting evidence regarding the degree of sulfomethylation and the multiplicity of the substitution of amine groups occurs. He et al. [16] have demonstrated that CMS is actually a mixture of various methanesulfonate derivatives. Differences have been observed in the content of four CMS brands that lead, after intravenous administration, to different plasma concentration-time profiles of the formed active colistin in rat plasma. Worth mentioning is that although the above-mentioned products were standardized by microbiological assays in vitro, the exposure to active colistin is different in vivo, emphasizing the need for CMS content standardization to achieve precise control over the drug’s bioavailability [17,18]. It should be noted that there is no analytical methodology for the quality control of CMS pharmaceutical products in the pharmacopoeia, partly because they were approved by the FDA in the late 1950s when control procedures were much less strict.

Our laboratory reported an ultra-high-performance liquid chromatography-mass spectrometry (UPLC–MS) methodology to determine the chemical characterization of CMS content in injectable formulations [19]. The aim of the presented study is the development of liquid chromatography coupled with an ultraviolet detector (UPLC-UV) to be applied as a quality-control procedure for CMS injectable formulations. This method is essentially different from the previous described UPLC–MS because the experimental design encompasses different a variable: wavelength against mass spectrometric peak. Furthermore, the mobile phase needs to be optimized against the UV signal because UV detection is affected in a vastly different way compared to mass spectrometry. It also has to be noted that the data treatment follows a completely different pipeline as the UV data are univariate and also collected by a low-resolution technique. Another step forward compared to UPLC–MS is the adoption of CMS peak ratios and the statistical treatment thereof. This method results in a unique metric that can be employed to characterize the quality of the tested CMS batch. 

Pharmaceutical companies employ mainly UV-based instrumentation, so the development of such a method was deemed useful for filling the gaps in the currently used microbiological assays, which are generally deemed to be of rather limited accuracy compared to chemical analysis methods. Metcalf et al. [20] reported an HPLC-UV method for a CMS assay in pharmaceutical aerosol samples, but only two chromatographic peaks of CMS were taken into account for the quantitation. In this study, all the chromatographic peaks corresponding to CMS compounds were taken into consideration for the assay of CMS in injectable formulations. The method was fully validated and applied to commercial batches to assess their content consistency. 

An effort was made to use freely available software so that the scientific community and pharmaceutical companies could have undisturbed access to the presented method for use as a CMS quality control procedure. The corresponding code can be found in the Supplementary Materials in order to be readily used.

## 2. Results and Discussion

### 2.1. Design of Experiments

CMS is an extremely complex mixture, so the optimized separation of its compounds was considered critical for unveiling the maximum possible number of underlying peaks and minimizing any matrix effects or interferences. Therefore, a Design-of-Experiments (DoE) strategy was followed, targeting the separation of the mixture components. It should be noted that Design-Expert 11 software from StatEase is not freely available; however, its use is not needed before each analysis. The proposed parameters are unaltered so it can be used without performing a DoE analysis before each analysis. 

In our previous published UPLC–MS method, an extensive optimization was performed for both the chromatographic and mass-spectrometric parameters. As the same UPLC instrument and analytical column were used for the current UPLC-UV method, it was deemed unnecessary to optimize the ratio of the organic modifier and the gradient elution program because a very good separation of CMS components had been already achieved using these parameters. In changing the detector from mass spectrometer to UV, it was deemed necessary to optimize the UV parameters. Thus, the ammonium formate concentration in the aqueous mobile phase was optimized, as it was found to affect the intensity of CMS chromatographic peaks, presumably because it presents UV absorbance in the near range. Therefore, the wavelength was also optimized since the baseline drift and the retention time changed with the concentration of the buffer and the CMS chromatographic peaks, which were eluted in different ratios of the two elution solvents. For the same reasons, the column temperature was deemed crucial for optimization, the goals of which were to obtain the highest resolution possible between the chromatographic peaks along with the highest sensitivity. The Box–Behnken design was preferred over other designs, such as the central composite and the three-level full-factorials designs, because it is more efficient at giving an accurate determination of the interactions between the variables [21]. Seventeen experiments including five center points were performed. Ammonium formate was tested at concentration levels 1 × 10^−3^, 2 × 10^−3^ and 3 × 10^−3^ M, wavelength at 214, 217 and 220 nm, while the temperature values were 29, 34.5 and 40 °C. There were two response factors: (1) the sum of the resolution between the peaks in each chromatogram and (2) the sum of the peak areas. Preferably, these numbers should be maximized, as the large resolution values indicated less overlapping and a higher sensitivity in the increased sum of areas. These values were calculated automatically using Empower software. The “ANalysis Of Variance” (ANOVA) showed that reduced cubic models (excluding all the uninformative interactions) could best fit the data, with *p*-values 0.0003 and 0.0002 for response factor 1 and 2 respectively, while the lack of fit was non-significant for both models. The repeatability (% Residual Standard Deviation, % RSD, *n* = 5) was excellent, with values 2.56% and 5.00% for the sum of resolutions and the sum of areas, respectively. The optimized conditions suggested by the obtained model were 1 × 10^−3^ M ammonium formate, 214 nm and 29 °C column temperature with a desirability value equal to 0.753. These conditions revealed 29 chromatographic peaks, while the corresponding number before the DoE experiments was 15.

### 2.2. Data Processing

#### 2.2.1. Data Pre-Processing

##### Baseline Correction

**Figure 1:**
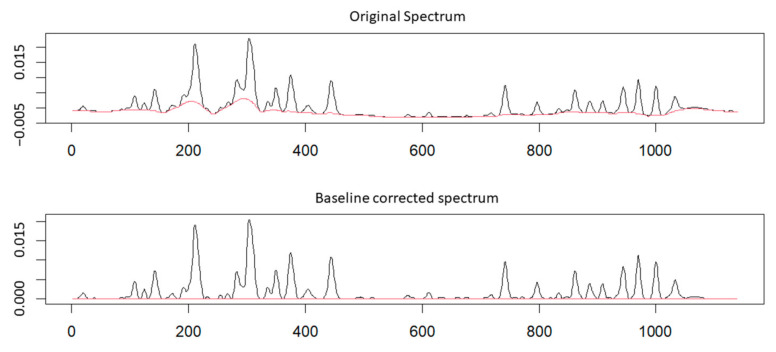
Original chromatogram and baseline corrected chromatogram after applying the “Iterative baseline correction algorithm based on mean” algorithm from “baseline” package in RStudio.

Even though the conditions for the analysis were optimized, chromatograms presented a high baseline due to the low detection wavelength. After applying several algorithms from the “baseline” package, the “Iterative baseline correction algorithm based on mean” was selected for the baseline correction. The criterion for choosing the “baseline” package parameters was the linearity of the calibration curve for each resolved CMS compound after application of the correction. The metric used for the choice of the algorithm and the corresponding parameters employed was the linearity function of each compound estimated by the correlation coefficient for each calibration curve. It should be noted that for the construction of the calibration curves that led to the determination of the baseline parameters, peak fitting, as described below, was also performed. The values of the parameters were set at 6 for the primary smoothing (lambda), 10 for the maximum number of iterations and 2000 for the number of buckets. Two values were tested for the half-width of local windows (hwi), 10 and 30. It was found that at the hwi setting of 30, 23 peaks were linear (R^2^ > 0.99), while that number was only 15 at the hwi setting of 10. The commands for the baseline correction are presented in Supplementary A.1. The original chromatogram and the chromatogram after the baseline correction are shown in Figure 1.

##### Peak Fitting and Integration

**Figure 2:**
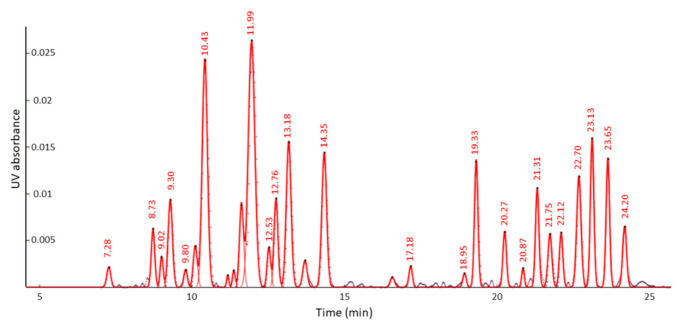
Chromatogram of 100 μg mL^−1^ CMS acquired by ultra-high-performance liquid chromatography coupled to a Waters ACQUITY UPLC™ Tunable UV (TUV) detector. 29 chromatographic peaks were discovered and integrated employing the free software Fityk (v. 1.3.1). The dataset points are presented by a dotted grey line.

Fityk was used to reveal, accurately describe and integrate the chromatographic peaks after the baseline correction. Peaks were added using the relative mode until the residuals were minimized in the corresponding plot, which indicated a good fitting (Supplementary B, Figure S1). Caution was taken to reveal and integrate the same peaks in all the acquired chromatograms. After processing, 29 peaks were discovered as is presented in Figure 2. It should be noted that in our previous UPLC–MS work, it was not possible to match the chromatographic peaks with the CMS components, due to the fact that a plethora of *m*/*z* signals were detected under each chromatographic peak perhaps because of source-induced dissociation. Thus, the chromatographic peaks of the current chromatogram acquired by UPLC–UV were not matched to the *m*/*z* values of CMS components.

#### 2.2.2. Data Analysis—Application to CMS Commercial Batches

As was stressed in our previous publication [19], the establishment of a CMS golden standard for the comparison of the batches is necessary for CMS quality control, which until now was based on microbiological assays, not on chemical content characterization. An ideal golden standard should exhibit high antimicrobial activity and low toxicity. Due to the fact that such a standard has not yet been established, the method development and validation presented here were performed by selecting a CMS batch arbitrarily as the reference.

The conformity of the CMS content (by means of the content of the respective forms in the formulation) was tested among different batches (*n* = 5; b1, b2, b3, b4 and b5) as well as within the same batch (*n* = 3; b4a, b4b and b4c). It should be noted that one of the tested batches had expired a year earlier (b5). Test solutions of 1 mg mL^−1^ for the reference and the tested CMS batches were prepared, diluted in LC–MS-grade water to a target concentration of 160 μg mL^−1^ and analyzed.

##### Univariate Model Construction

Linear models for each CMS peak were constructed using the Linear Regression platform of MEPHAS after importing the areas of the integrated peaks as comma-separated values (CSV) files (Supplementary A.2) After construction of linear regression models for the 29 chromatographic peaks, it was observed that 23 presented a correlation coefficient of R^2^ > 0.99; thus, only these linear models were finally kept. The best fit values of the intercept (B0) and of the coefficient of the linear term (B1), as well as the standard errors associated to the coefficients, the R^2^ and the standard error of estimate (Sy.x) for the 23 univariate models are presented in Table 1. Peaks were named based on their retention times. The values of the linear terms cannot be explained on the basis of the CMS structure as these still remain unknown. However, it can be observed that CMS components with UV absorbance values > 0.01 (Figure 2) presented B0 in the range of 10^−5^, while peaks with lower absorbance had B0 in the range of 10^−6^. The differences in the absorbance could mainly be attributed to the amount of these components in the CMS content. The interpolated values for the tested batches are presented in Table 2. The upper and lower limits of the interpolated values were also calculated at a 95% confidence interval (Supplementary B, Table S1).

In the 5-year-old batch (b5) most of the peaks were higher than those in the younger batches. However, there were also peaks that were much lower compared to the other tested batches. As the structure of CMS components is unknown, it was not clear why this was happening. An explanation is that some CMS components underwent degradation (e.g., peak_8.73, peak_10.43, peak_23.13, peak_23.65), thus resulting in lower intensities. On the other hand, the resultant components combined with those already existed in the CMS content, led to increased intensities (e.g., peak_9.3, peak_12.76). Another reason is the different degree of substitution of the CMS components that could have occurred during the manufacturing of the pharmaceutical products 5 years ago. The presented work is very useful for exactly this reason: to show the possible inconsistencies of the batches owing to different degrees of substitution of CMS components that eventually led to different bioavailability. The stock solution (1 mg mL^−1^) of the b5 batch as well as of all batches was freshly weighted and diluted with accuracy, so the differences in the content of b5 was not attributed to wrong handling.

##### Multivariate Model Construction

A Partial Least Squares regression (PLSr) model was constructed employing the Partial Least Squares Regression platform of MEPHAS, importing the file with the areas of the 29 chromatographic peaks (Supplementary A.2). The CMS peak areas were used as the independent variables (X), whereas the levels of CMS were the dependent (Y). The leave-one-out cross-validation and the SIMPLS algorithm were selected for the PLSr analysis. The optimized number of components was determined by the construction of models with 1, 2, 3 and 4. The mean square error of prediction (MSEP) and the root mean square error of prediction (RMSEP) were high at the first component, whereas they were much lower even for the second component addition. The R^2^ was 0.997 using the first component. Thus, only the first component was kept for the PLSr model, as it was adequate for the model description (Supplementary B, Figure S2). The absolute % error of the predicted values was <1.35%. The areas of the tested batches were also analyzed to calculate the amount of CMS using the PLSr model. The interpolated values of the CMS batches employing the PLSr are shown in Table 3.

It should be emphasized that the PLSr model provided a total estimation of the CMS amount without paying attention to the ratio of the CMS component; that is, if some components changed signal mutually but equally, then the model was not able to discriminate.

##### Validation of the Univariate and Multivariate Models

The validation was performed for the 23 linear regression models and the PLSr model. Since, in the literature, there are no consensus validation procedures that regulate the performance quality characteristics of multivariate models, the regulatory requirements ICH Q2(R1) were adopted for both univariate and multivariate models in the current study. Linearity was tested over the range of 100–220 μg mL^−1^. Accuracy expressed as % standard error from the nominal value (% E) was assessed by analyzing samples at three concentration levels (130, 160 and 190 μg mL^−1^) and at three analytical runs. All the univariate models exhibited accuracy lower than 2.40% at the three tested levels, whereas for the PLSr model the accuracy was lower than 2.83%. Repeatability (the precision under the same operating condition over a short internal of time) and intermediate precision (the variations between different analytical days, *n* = 3) were expressed as % relative standard deviation (%RSD). The PLSr model presented better precision than the univariate models. Stability was examined at the same levels injecting the same sample (*n* = 3) every three hours (autosampler stability). The results from both univariate and multivariate validation showed that the CMS components were stable at the duration of the data acquisition. The robustness was examined at 160 μg mL^−1^ by making deliberate changes (+/−5%) of the starting percentage of the organic modifier and the column temperature. The limit of detection (LOD) and limit of quantitation (LOQ) were calculated only for the univariate models but not for the multivariate model because a multivariate calibration equation is not necessarily similar for all the contributing variables. The LOD and LOQ values were assessed using the equations 3.3 × σ/slope and 10 × σ/slope, respectively, and they were found to range from 4.12 to 17.98 and 12.48 to 54.49, respectively. Before the experiments, the instrumental performance expressed as % RSD was also tested by injection of the reference sample (*n* = 3) and it was found to be <3.4%. Therefore, the instrumental performance exhibited only a small contribution to the overall variabilities of the validation procedure. Table 4 presents the summary of the validation results for the 23 linear regression models and the PLSr model.

##### Principal Component Analysis

In order to compare the content of the batches on the basis of the CMS component ratios, it was decided to divide all peaks with a “reference” peak. Thus, peak_14.42 was selected because it showed no overlap in the chromatogram, and its linear regression model had the highest correlation coefficient (R^2^ = 0.9996). Thus, the areas of the 23 peaks that exhibited R^2^ > 0.99 were divided by the area of peak_14.42. The same procedure was performed for the tested batches.

**Figure 3:**
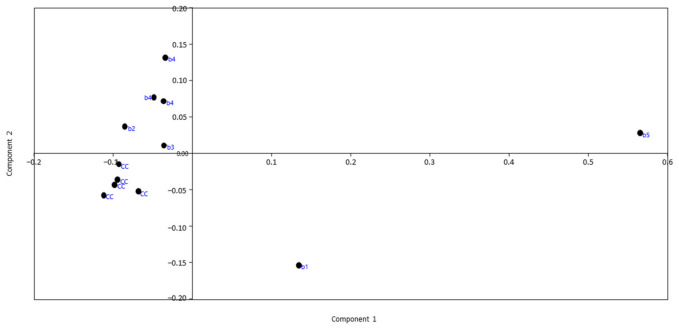
The score scatter plot (PC1 vs PC2) of the Principal Component Analysis (PCA) model constructed by employing the PAST free software for the calibration curve points (CC) and the CMS batches b1, b2, b3, b4 and b5. The data were the area ratios of the 23 chromatographic peaks divided by the peak_14.42.

A Principal Component Analysis (PCA) model was constructed employing the Principal Component Analysis platform of MEPHAS, importing the peak area ratios (calibration points and batches) as a CSV file (Supplementary A.2). PAST software was also used for PCA (Supplementary A.3), because MEPHAS presented the limitation that the number of variables should be less than the number of samples. Both software gave the same results. Four principal components (PC) were found to describe the model adequately (Supplementary B, Figure S3), with R2X and Q2 equal to 0.992 and 0.616, respectively. The 1st PC held 79.7% of the variance, and the 2nd PC held 12.8%. Therefore, the main differences were summarized for the first two PCs holding more than 92% of the total variability. After a 7-fold bootstrapping the eigenvalues were 83.0% for the 1st PC and 24.0% for the 2nd PC (Supplementary B, Table S2). The scores scatter plot of PC1 vs PC2 is presented in Figure 3. It was observed that the calibration curve (CC) points were at the same cluster as these samples were prepared from the same reference batch. The batches b2, b3 and b4 were similar with that of the calibration curve, whereas b1 and b5 were markedly different. As it can be observed, the projection of the batches on the 1st PC (which holds the main part of the variability) lied together, while the b5 was clearly separate. The projection in PC2 rendered b1 as also clearly distinguishable from the other batches. The batch b5 was an outlier, indicating that the content of this batch was different from the others. It should be noted that the distances between the inter-batch distance of the CC points was similar to that of the bache 4 which was analyzed in triplicate, indicating visually the repeatability of the method. Overall, PCA could discern out-of-specification batches (e.g., batch b5 was expired), but also microbiologically assayed batches that were different from the individual CMS components (e.g., batch b1). PCA involves the initial rotation of the matrix according to the highest variability observed. This could lead to less accurate results compared to the univariate analysis. Therefore, the univariate analysis was also followed.

##### Similarity Tests

The above-mentioned peak ratios of the tested batches were also compared with the reference ones by employing several similarity tests embedded in the “proxy” package. The table was imported to RStudio as a CSV file. The commands that were followed for the computation of the similarity tests are presented in Supplementary A.4. The performed similarity measures were: eJaccard, cosine, eDice, correlation and gower, while the performed distance measures were: bray, canberra, chord, divergence, euclidean, geodesic, hellinger, kullback, manhattan, podani, soergel, supremum, whittaker and bhjattacharyya. The results are presented in Table 5. It was indicated that batch b5 was different from the reference batch as it presented the lower value at all the tests.

##### Heatmaps and Structure Similarity Index

**Figure 4:**
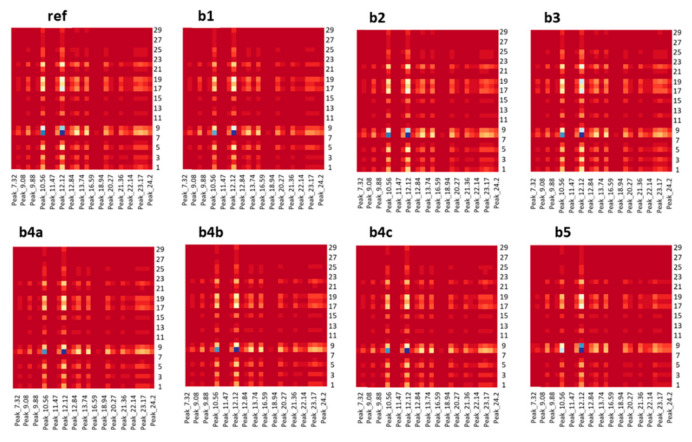
Heatmaps for the peak ratios in the reference batch and the batches b1, b2, b3, b4 (a, b, c) and b5 that were produced using the “stats” package in RStudio. Low and high extreme values are emphasized with dark colors, while light colors represent middle values.

Due to the fact that the division of the peak areas by a particular peak could have led to uncertainty because one variable (i.e., the peak 14_42) could leverage the analysis, one more step was followed to explore the CMS content further. All peaks were divided by all the peaks, resulting to a square matrix for each sample. The resulting matrices could be visually compared afterwards as heatmaps. The heatmaps of the reference batch and the tested batches are presented in Figure 4. As a visual inspection suffers from high bias and low accuracy, it was deemed crucial to depict the similarity between the reference heatmap and the tested ones using a simple-to-interpret metric. Thus, the heatmaps were compared using the “SPUTNIK” package, which employs the structural similarity index (SSIM). This index is widely used for evaluating image quality [22] and it has also been applied to the evaluation of chromatographic procedures [23]. The results are presented in Table 6. Batch b5 had a low SSIM value, indicating the content inconsistency of that batch compared to the reference, which was in agreement with the PCA results. The commands for the “stats” and “SPUTNIK” packages are presented in Supplementary A.5.

## 3. Materials and Methods

### 3.1. Chemicals

All CMS samples were from the same brand and purchased from a drug store. LC–MS-grade acetonitrile and formic acid were purchased from Carlo Erba reagents (Val de Reuil Cedex, France). LC–MS-grade methanol was obtained from Fisher Scientific (Loughborough, U.K.). Ammonium formate was purchased from Sigma-Aldrich (Steinheim, Germany). Ultrapure water was produced by a Millipore Direct-Q System (Molsheim, France).

### 3.2. Instrumentation

The instrumentation consisted of a Waters ACQUITY UPLC (Milford, MA, USA) module equipped with a binary solvent manager system and an autosampler thermostatically controlled at 4 °C coupled to a Waters ACQUITY UPLC™ Tunable UV (TUV) detector. The analysis was performed on a Waters Acquity BEH C_8_ (2.1 mm × 100 mm, 1.7 μm) (Milford, MA, USA) analytical column. After optimization, the chromatographic conditions were; aq. ammonium formate 0.001 M as solvent A and methanol/acetonitrile 79/21 (*v*/*v*) as solvent B. The column temperature was maintained at 29 °C. The gradient mode was the same as the previously described program by our laboratory [19]. Briefly, the elution started with 15% B and was programmed to reach 80% B in 34 min at a flow rate of 0.15 mL min^−1^. The UV detector was set at 214 nm. The sample injection volume was 10 μL. Instrumental settings, data acquisition and processing were controlled via Empower 2 software.

### 3.3. Sample Preparation

Stock solutions (1 mg mL^−1^) of CMS were prepared in methanol and stored at 30 °C. Calibration standards of CMS were freshly and independently prepared 3 times from the stock; that is, they were weighted and diluted appropriately in water in order to reach concentration levels of 100, 130, 160, 190, 220 μg mL^−1^, which corresponded to 80–120% of the theoretical amount of 160 mg CMS in formulations.

For the tested batches (b1, b2, b3, b4a, b4b, b4c and b5), a stock solution of 1 mg mL^−1^ was prepared for each tested batch by weighting the total CMS and thereafter dilution was performed in order to reach a concentration level of 160 μg mL^−1^.

### 3.4. Design of Experiments

Design of Experiments (DoE) for the chromatographic condition of CMS assay was performed via Design-Expert 11 software from StatEase. The Box–Behnken experimental design for response surface methodology was selected for the optimization of the column temperature, the ammonium formate concentration in an aqueous mobile phase and the wavelength, and it performed 17 runs that included 5 center points.

### 3.5. Data Processing

**Figure 5:**
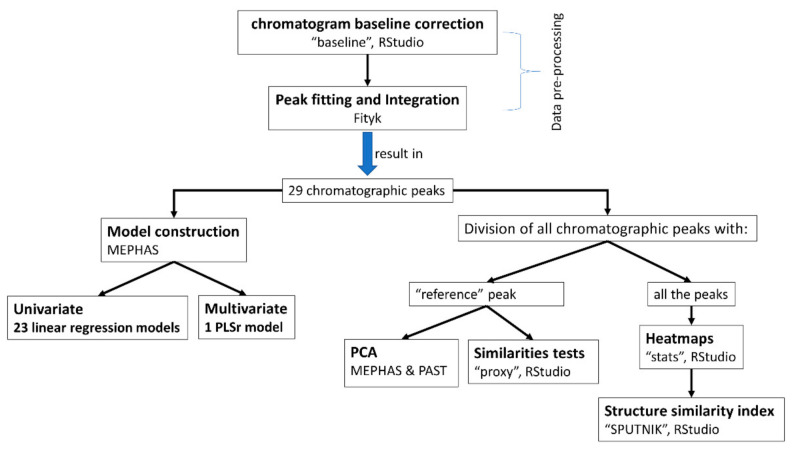
Flowchart of the analytical procedures for the assessment of CMS in formulations. Rstudio, Fityk, MEPHAS and PAST are the freely available software used.

The steps followed in the present study are summarized in Figure 5. The freely available software that was employed for method development is presented in Table 7.

#### 3.5.1. Baseline Correction

The baseline of the chromatograms was corrected using the free and open-source software RStudio, which uses R programming language. The baseline correction was performed using the “baseline” package. The raw data were exported from Empower as CDF files. They were converted to CSV files and uploaded to RStudio as a matrix, where the first row of the matrix included the time and the second row the intensities. After the baseline correction using the appropriate algorithm and parameters, RStudio returned a CSV file with the corrected intensities.

#### 3.5.2. Peak Fitting and Integration

The free software Fityk (version 1.3.1) was used for peak fitting after the baseline correction and importing the files produced by RStudio. The exported results were converted to CSV files.

#### 3.5.3. Univariate and Multivariate Data Analysis

MEPHAS, a free and open-source web-based interactive GUI [24], was used for the univariate (linear regression) and multivariate (Partial Least Squares regression–PLSr and Principal Component Analysis–PCA) for the CMS data analysis. PCA was also performed using PAST software [25].

#### 3.5.4. Similarity Measures and Structure Similarity Index

The similarity and distance measures were assessed employing the “proxy” package in RStudio. Heatmaps were constructed using the “stats” package, and they were compared employing the “SPUTNIK” package that provides the calculation of the structure similarity index (SSIM) between two heatmaps.

### 3.6. Method Validation

The univariate and multivariate models were validated examining linearity, precision (repeatability and intermediate precision), accuracy, reproducibility, stability, robustness, limit of detection (LOD) and limit of quantitation (LOQ) according to the ICH Q2(R1) analytical procedure guidelines (https://www.ema.europa.eu/en/documents/scientific-guideline/ich-q-2-r1-validation-analytical-procedures-text-methodology-step-5_en.pdf, accessed on 19 February 2021).

## 4. Conclusions

In the current study, a UPLC–UV methodology was developed for the quality control of CMS in commercial formulations using freely available software. DoE optimization of the chromatographic parameters resulted in an adequate separation of the CMS components stemming from an extremely complex prodrug mixture. A multivariate PLSr model was developed to assess the total amount of CMS in pharmaceutical formulations and 23 univariate linear regression models to determine each CMS compound separately. All the models were fully validated. The concentration range was lower than that reported by Metcalf et al. (100–220 μg mL^−1^ instead of 0.05–7 mg ml^−1^). The linear regression models were constructed to measure the concentration of each CMS component. It is true that there are no calibration standards for the CMS components; thus, a CMS batch was selected arbitrarily to serve as the reference batch. The components of that reference batch were the calibration standards for the construction of the linear models. The advance chemometrics employed in the presented work gave an opportunity to access the ratios of the CMS components in the batches and to depict the outlier batches by one number. The 23 linear regression models were constructed in order to measure the concentration of each CMS component in the batches. The ratios of the peaks were not always the same, and this is the reason that all peaks were divided by all the peaks, resulting in squared matrices that were compared as heatmaps using the structure similarity index. Thus, three criteria should be met. The sum amount should be the similar; the amount of every peak should be at a predefined level; and the ratios among the substances should be also clearly defined. This ensured that the overall composition was the same; therefore, the bioavailability and the biological activity are clearly defined.

This is the first simple, low-cost UPLC–UV methodology whatsoever for the direct chemical quantitation of CMS content based on all chromatographic peaks. The method was applied to determine the constitution of CMS in pharmaceutical formulations by examining different commercially available batches. The batchs’ analysis revealed that no great chemical inconsistencies were observed among the tested samples except the expired batch, which had different chemical content from the reference batch.

It should be noted that the reference CMS batch was chosen arbitrarily in order to perform the batch comparison. Thus, there is an immediate need for a gold standard characterized by high antimicrobial activity and low toxicity to be established. Using this method we aimed to devise a methodology that can be used as a tool for the industry and the regulatory bodies to set up a gold-standard procedure for the proper chemical determination of this substance. In the absence of one, we chose one of the batches to serve as standard and showed that once such a gold standard is created, which could easily be done now with this method, a validation pipeline for the producers of the API as well as pharmaceutical substances could be created. Briefly, one batch was used arbitrarily as a standard (in the future there will be a standard created and provided by the regulatory bodies) and we were able to show that, using this arbitrary standard, we could definitely validate the CMS drug properly. Applying the presented methodology widely, quality control of CMS batches can be performed and quantitating each chromatographic peak, thus leading to injectable formulations showing better control of the desired bioavailability. Furthermore, the separation and quantification of all major chromatographic peaks could aid in the structural elucidation of the compounds consisting of this very complex mixture. Finally, the shelf-life estimate on the basis of chemical assessment can be performed, which leads to reliable results for the homogeneity of the sample content.

## Figures and Tables

**Table 1:** The best-fit values B0 and B1, the standard errors associated to the coefficients, the correlation coefficient (R^2^) and the standard error of the estimate (Sy.x) of the 23 linear models for the CMS peaks, as they were calculated by the MEPHAS software.

No	Name	Best-Fit Values		Std. Error		R^2^	Sy.×
		B0	B1	B0	B1		
1	Peak_7.28	1.13 × 10^−5^	1.73 × 10^−6^	4.18 × 10^−6^	2.52 × 10^−8^	0.9994	2.39 × 10^−6^
2	Peak_8.73	−2.6 × 10^−5^	4.88 × 10^−6^	1.99 × 10^−5^	1.2 × 10^−7^	0.998	1.14 × 10^−5^
3	Peak_9.02	4.36 × 10^−5^	1.73 × 10^−6^	1.55 × 10^−5^	9.33 × 10^−8^	0.991	8.85 × 10^−6^
4	Peak_9.30	0.000105	8.27 × 10^−6^	2.82 × 10^−5^	1.7 × 10^−7^	0.998	1.61 × 10^−5^
5	Peak_9.80	3.06 × 10^−5^	1.32 × 10^−6^	7.07 × 10^−6^	4.27 × 10^−8^	0.997	4.05 × 10^−6^
6	Peak_10.43	0.000177	2.96 × 10^−5^	1.29 × 10^−4^	7.8 × 10^−7^	0.998	7.4 × 10^−5^
7	Peak_11.99	3.45 × 10^−5^	3.62 × 10^−5^	1.45 × 10^−4^	8.79 × 10^−7^	0.998	8.34 × 10^−5^
8	Peak_12.53	2.0 × 10^−6^	3.0 × 10^−6^	6.83 × 10^−6^	4.13 × 10^−8^	0.9994	3.92 × 10^−6^
9	Peak_12.76	1.29 × 10^−4^	7.74 × 10^−6^	1.69 × 10^−5^	1.02 × 10^−7^	0.9995	9.71 × 10^−6^
10	Peak_13.18	2.35 × 10^−5^	1.61 × 10^−5^	3.82 × 10^−5^	2.31 × 10^−7^	0.9994	2.19 × 10^−5^
11	Peak_14.35	−3 × 10^−5^	1.54 × 10^−5^	4.29 × 10^−5^	2.59 × 10^−7^	0.9996	2.46 × 10^−5^
12	Peak_17.18	1.19 × 10^−5^	1.59 × 10^−6^	5.98 × 10^−6^	3.61 × 10^−8^	0.998	3.43 × 10^−6^
13	Peak_18.95	1.11 × 10^−5^	1.18 × 10^−6^	5.73 × 10^−6^	3.46 × 10^−8^	0.997	3.28 × 10^−6^
14	Peak_19.33	1.02 × 10^−4^	1.07 × 10^−5^	2.4 × 10^−5^	1.45 × 10^−7^	0.9994	1.38 × 10^−5^
15	Peak_20.27	6.23 × 10^−5^	4.29 × 10^−6^	1.12 × 10^−5^	6.79 × 10^−8^	0.9993	6.44 × 10^−6^
16	Peak_20.87	3.0 × 10^−6^	1.29 × 10^−6^	1.23 × 10^−5^	7.41 × 10^−8^	0.990	7.03 × 10^−6^
17	Peak_21.33	−3.8 × 10^−5^	8.46 × 10^−6^	2.03 × 10^−5^	1.23 × 10^−7^	0.9994	1.16 × 10^−5^
18	Peak_21.75	−3.2 × 10^−5^	4.77 × 10^−6^	2.36 × 10^−5^	1.43 × 10^−7^	0.997	1.35 × 10^−5^
19	Peak_22.12	1.63 × 10^−5^	3.82 × 10^−6^	1.44 × 10^−5^	8.68 × 10^−8^	0.998	8.24 × 10^−6^
20	Peak_22.70	−9.4 × 10^−5^	1.13 × 10^−5^	2.47 × 10^−5^	1.49 × 10^−7^	0.9995	1.41 × 10^−5^
21	Peak_23.13	−4.7 × 10^−7^	1.24 × 10^−5^	3.11 × 10^−5^	1.88 × 10^−7^	0.9993	1.78 × 10^−5^
22	Peak_23.65	−6.4 × 10^−5^	1.08 × 10^−5^	3.77 × 10^−5^	2.28 × 10^−7^	0.998	2.16 × 10^−5^
23	Peak_24.20	4.26 × 10^−4^	3.72 × 10^−6^	3.07 × 10^−5^	1.86 × 10^−7^	0.992	1.76 × 10^−5^

**Table 2:** Interpolated values (μg mL^−1^) of the CMS compounds for the tested and the reference batches as they were calculated by the linear regression models developed in MEPHAS. The peaks that presented % Error > 20.0 from the reference value are bolded.

Peaks	Batches							
	Reference	b1	b2	b3	b4a	b4b	b4c	b5
Peak_7.28	161.38	172.37	160.18	177.90	**197.40**	**203.45**	183.76	**215.34**
Peak_8.73	160.29	166.43	159.52	186.13	**195.02**	**203.04**	185.78	151.79
Peak_9.02	163.82	155.89	159.66	163.45	159.12	164.16	152.44	151.37
Peak_9.30	159.03	165.36	159.00	164.58	176.96	186.60	172.23	**222.71**
Peak_9.80	157.88	**123.53**	158.68	**216.73**	174.85	183.56	170.11	**219.82**
Peak_10.43	162.62	151.30	161.01	167.91	173.71	180.11	164.75	148.58
Peak_11.99	162.82	167.38	161.37	175.33	179.80	187.03	171.63	**206.85**
Peak_12.53	160.67	159.21	157.64	174.67	180.31	189.25	171.96	162.05
Peak_12.76	160.98	153.46	156.65	162.76	169.68	176.89	159.61	**200.00**
Peak_13.18	158.58	145.82	157.86	166.52	171.98	178.42	162.42	155.72
Peak_14.35	159.22	154.64	158.66	169.20	176.24	182.93	166.23	173.91
Peak_17.18	157.61	150.79	171.50	188.85	**208.18**	**218.25**	**198.88**	165.47
Peak_18.95	160.51	129.14	168.93	173.40	178.19	182.98	189.46	143.35
Peak_19.33	159.68	141.66	168.03	174.95	191.18	**197.33**	186.53	166.00
Peak_20.27	159.49	141.25	165.80	174.65	182.64	**191.58**	179.32	**234.93**
Peak_20.87	162.79	172.14	172.45	184.84	**214.98**	**226.10**	**213.74**	**224.00**
Peak_21.33	159.86	158.55	169.28	178.59	**196.83**	**204.21**	**192.78**	**200.75**
Peak_21.75	157.82	144.83	166.24	174.96	**191.92**	**197.88**	185.96	**194.90**
Peak_22.12	159.21	140.39	166.33	166.10	179.57	186.70	175.73	**239.08**
Peak_22.70	158.57	147.71	166.63	170.62	186.55	**194.49**	183.81	**211.31**
Peak_23.13	157.88	133.52	165.97	173.85	183.17	188.17	178.13	134.49
Peak_23.65	157.18	130.46	163.74	168.55	180.30	185.57	173.56	137.05
Peak_24.20	167.26	166.95	176.09	179.92	198.30	**206.27**	193.16	**234.58**

**Table 3:** Interpolated values and the confidence intervals of Y (jack–knifed) for the tested batches as they were calculated by the developed PLS-R model employing the free software MEPHAS. The % errors from the theoretical value of 160 μg mL^−1^ are also presented.

Batch	Interpolated Values CMS (μg mL^−1^)	Upper Limit	Lower Limit	% Error
b1	156.01	150.29	151.67	−2.49
b2	161.85	163.82	164.08	1.15
b3	171.61	174.73	176.06	7.25
b4a	178.50	183.63	185.61	11.56
b4b	185.21	190.63	193.21	15.75
b4c	170.72	178.04	179.46	6.70
b5	179.33	185.90	187.42	12.08

**Table 4:** Accuracy, repeatability, intermediate precision, stability and robustness assessed for the 23 univariate linear regression models and the Partial Least Square regression (PLSr) model developed for the assay of CMS in pharmaceutical formulations using the proposed UPLC–TUV methodology. The limit of detection (LOD) and limit of quantitation (LOQ) values were calculated only for the univariate model.

23 Univariate Linear Regression Models						
Concentration Level (μg mL^−1^)	Accuracy (*n* = 3, %E)	Precision (*n* = 3, %RSD)		Stability (*n* = 3, %RSD)	Robustness (*n* = 3, %RSD)	
		Repeatability	Intermediate Precision			
130	<4.78	<2.34	<3.50	<2.75		
160	<4.13	<2.40	<3.35	<2.15	<3.14	
190	<3.35	<2.28	<2.95	<2.37		
	LOD (μg mL^−1^)	LOQ (μg mL^−1^)				
	4.12–17.98	12.48–54.49				
**Multivariate PLSr Model**						
**Concentration Level (μg mL^−1^)**	**Accuracy (*n* = 3, %E)**	**Precision (*n* = 3, %RSD)**		**Stability (*n* = 3, %RSD)**	**Robustness (*n* = 3, %RSD)**	
		**Repeatability**	**Intermediate Precision**			
130	2.83	0.28	1.13	1.75		
160	0.64	0.45	0.99	1.26	<2.64	
190	1.17	0.57	1.24	1.38		

**Table 5:** Results of the similarity and distance measures that were performed employing the “proxy” package in RStudio. The CMS peak ratios of the tested batches b1, b2, b3, b4 (a, b, c) were compared with those of the reference batch.

Tests	Batches						
	b1	b2	b3	b4a	b4b	b4c	b5
Similarity measures							
ejaccard	0.995	0.999	0.999	0.999	0.999	0.997	0.972
cosine	0.997	1.000	1.000	0.999	0.999	0.999	0.986
eDice	0.997	1.000	1.000	0.999	0.999	0.999	0.986
correlation	0.996	1.000	0.999	0.999	0.999	0.998	0.974
Gower	0.693	0.855	0.795	0.707	0.695	0.612	0.236
Distance measures							
Bray	0.967	0.987	0.985	0.980	0.980	0.973	0.923
Canberra	0.540	0.737	0.668	0.608	0.602	0.532	0.349
Chord	0.933	0.983	0.973	0.966	0.966	0.958	0.859
divergence	0.953	0.991	0.979	0.967	0.966	0.949	0.845
Euclidean	0.782	0.911	0.898	0.878	0.877	0.836	0.601
Geodesic	0.933	0.983	0.973	0.966	0.966	0.958	0.859
Hellinger	0.962	0.988	0.981	0.976	0.975	0.972	0.920
Kullback	0.997	1.000	0.999	0.999	0.999	0.998	0.985
Manhattan	0.544	0.748	0.724	0.663	0.662	0.581	0.319
Podani	0.975	0.983	0.967	0.943	0.943	0.943	0.878
Soergel	0.755	0.878	0.866	0.827	0.827	0.773	0.570
supremum	0.837	0.962	0.924	0.938	0.938	0.918	0.693
Whittaker	0.969	0.990	0.986	0.982	0.982	0.978	0.924
Bhjattacharyya	0.875	0.944	0.934	0.913	0.913	0.886	0.760

**Table 6:** Structure similarity index (SSIM) values between the reference heatmap of the CMS peak ratios and the heatmaps of the tested batches (b1, b2, b3, b4a, b4b, b4c and b5), as they were calculated by the “SPUTNIK” package in RStudio. Batch b5 had the lowest value.

Batch	SSIM
b1	0.961
b2	0.998
b3	0.994
b4a	0.960
b4b	0.961
b4c	0.958
b5	0.918

**Table 7:** The freely available software employed for the analysis of CMS.

Software		Scope	Available on
Rstudio			https://rstudio.com/ (accessed on 19 February 2021)
packages:	“baseline”	chromatogram baseline correction	https://cran.r-project.org/web/packages/baseline/index.html (accessed on 19 February 2021)
	“proxy”	similarity and distance measures	https://cran.r-project.org/web/packages/proxy/index.html (accessed on 19 February 2021)
	“stats”	heatmaps	https://www.rdocumentation.org/packages/cran.stats/versions/0.1 (accessed on 19 February 2021)
	“SPUTNIK”	structure similarity index	https://cran.r-project.org/web/packages/SPUTNIK/index.html (accessed on 19 February 2021)
Fityk		Peak fitting and integration	https://fityk.nieto.pl/ (accessed on 19 February 2021)
MEPHAS		linear regression model, PLSr, PCA	https://alain003.phs.osaka-u.ac.jp/mephas/ (accessed on 19 February 2021)
PAST		PCA	https://past.en.lo4d.com/windows (accessed on 19 February 2021)

## Data Availability

The data presented in this study are available in this article and supplementary material.

## Supplementary Materials

The following are available online: Supplementary A Figure S1: Part of the table uploaded to MEPHAS for the linear regression models, Figure S2: MEPHAS interface of the data preparation for the Partial Least Squares regression model, Figure S3: MEPHAS interface for the building of Partial Least Squares regression model, Figure S4: Part of the table uploaded to MEPHAS for the principal component analysis, Figure S5: MEPHAS interface of the data preparation for the Principal Component Analysis, Figure S6: Imported data to the PAST software, Figure S7: Part of the table imported to RStudio in order to perform the similarities tests using the “proxy” package. Supplementary B; Figure S1: Peak fitting in the UPLC–UV chromatogram of 100 μg mL−1 CMS, employing the Fityk (v. 1.3.1) software. The residual plot above the chromatogram indicates the good fitting, Figure S2: The values of Mean square error of prediction (MSEP), Root mean square error of prediction (RMSEP) and R2 by using 1–4 components for PLSr model construction. It is observed that one component can fully describe the model, Figure S3: Scree plot as it is produced by PAST software. Table S1: Interpolated values (mg mL−1) of the CMS components in the tested batches, as they were calculated by the linear regression models developed in MEPHAS. The upper and lower limits of the interpolated values at the 95% confidence level are also presented, Table S2: Eigenvalues, % variances, Eig 2.5% and Eig 97.5% of the principal components after 7-fold bootstrapping as calculated from PAST software during the Principal Component Analysis.
