# Supplementary material for: A Novel Validated Injectable Colistimethate Sodium Analysis Combining Advanced Chemometrics and Design of Experiments
Source: Molecules. 2021 Mar 11;26(6):1546. doi: 10.3390/molecules26061546 (PMC8000333; doi:10.3390/molecules26061546)
Supplement: Supplementary file 1 [file molecules-26-01546-s001.zip › molecules-1135428-supplementary-final/Supplementary aí.docx]

**Supplementary A**

1. **Baseline correction**

> library("baseline")

> run1 <- read_delim("C:/Temp/data_run1.csv", ";", escape_double = FALSE, trim_ws = TRUE)

> run1_matrix <- as.matrix(run1)

> baselineGUI(run1_matrix)

Select a GUI toolkit

1: gWidgets2RGtk2

2: gWidgets2tcltk

Selection: 2


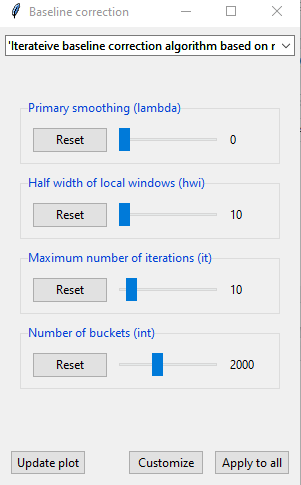


A new window appears where the appropriate algorithm and parameters can be selected. By pressings the buttons “Update plot” and “Apply to all”, the baseline corrected chromatograms can be exported by pressing “Apply and export” in the following window:


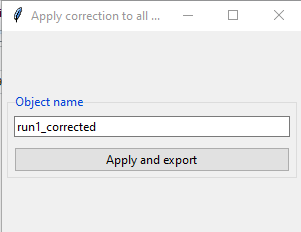


Data can be exported as .CSV files:

> tran <- t(run1_corrected@corrected)

> write.csv([tran, file="run1_corrected.csv](mailto:corrected.spectra@corrected,file=%22spectra.csv)")

1. **MEPHAS**

**Linear regression model**

The areas of the integrated peaks were imported as .CSV files. The uploaded file was a matrix including the names of peaks in the first raw and the levels of the calibration curve in the last column. The first column included a serial number starting from 1 (**Figure S1**). The appropriate separator (i.e. comma, tab, semicolon) was selected in order to read the file correctly.

**Figure S1**. Part of the table uploaded to MEPHAS for the linear regression models.

**Partial Least Squares regression**

The areas of the integrated peaks were imported as .CSV files. The uploaded file was a matrix including the names of peaks in the first raw and the levels of the calibration curve in the last column. The first column included a serial number starting from 1. The appropriate separator (i.e. comma, tab, semicolon) was selected in order to read the file correctly (**Figure S2**). Then we press the button “Go to build PLSR Model”. We choose the dependent variables (Y), the independent variables (X) and the number of components (**Figure S3**). We see the results by pressing the button “Show Results”. We also upload the data of the unknown samples as .CSV file with the same format of the model data. We press the button “Show Prediction” in order to see the results of the unknown samples.

**
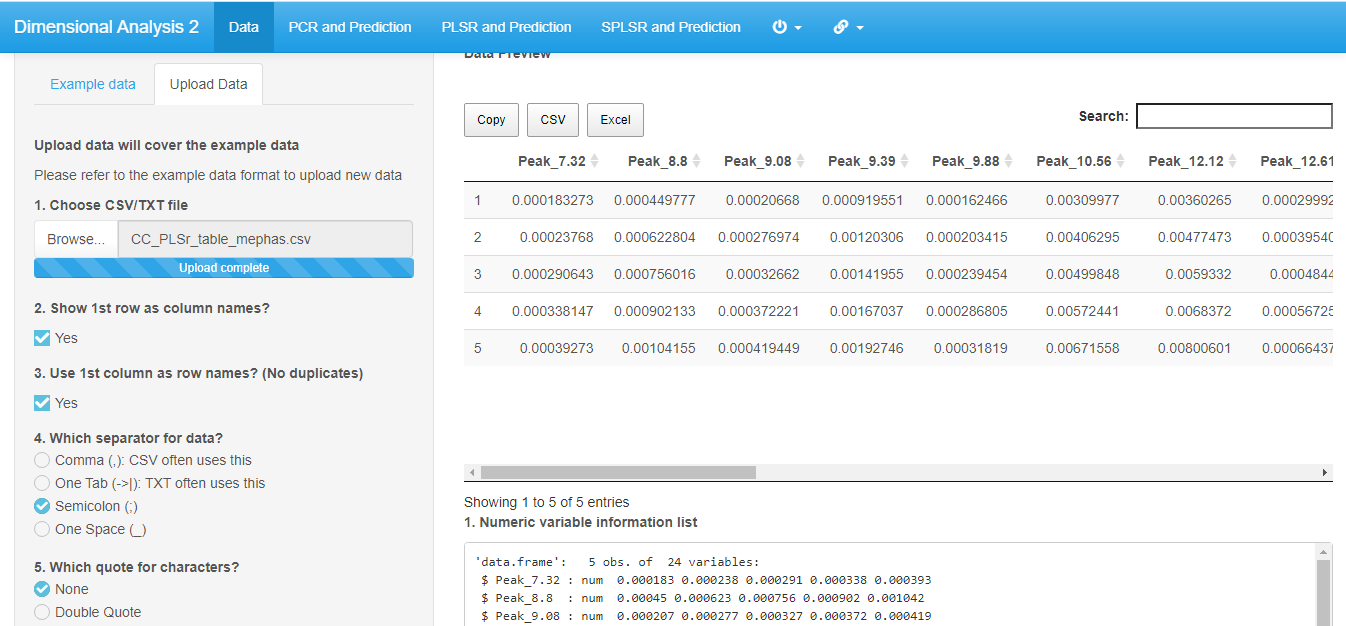
**

**Figure S2.** MEPHAS interface of the data preparation for the Partial Least Squares regression model.


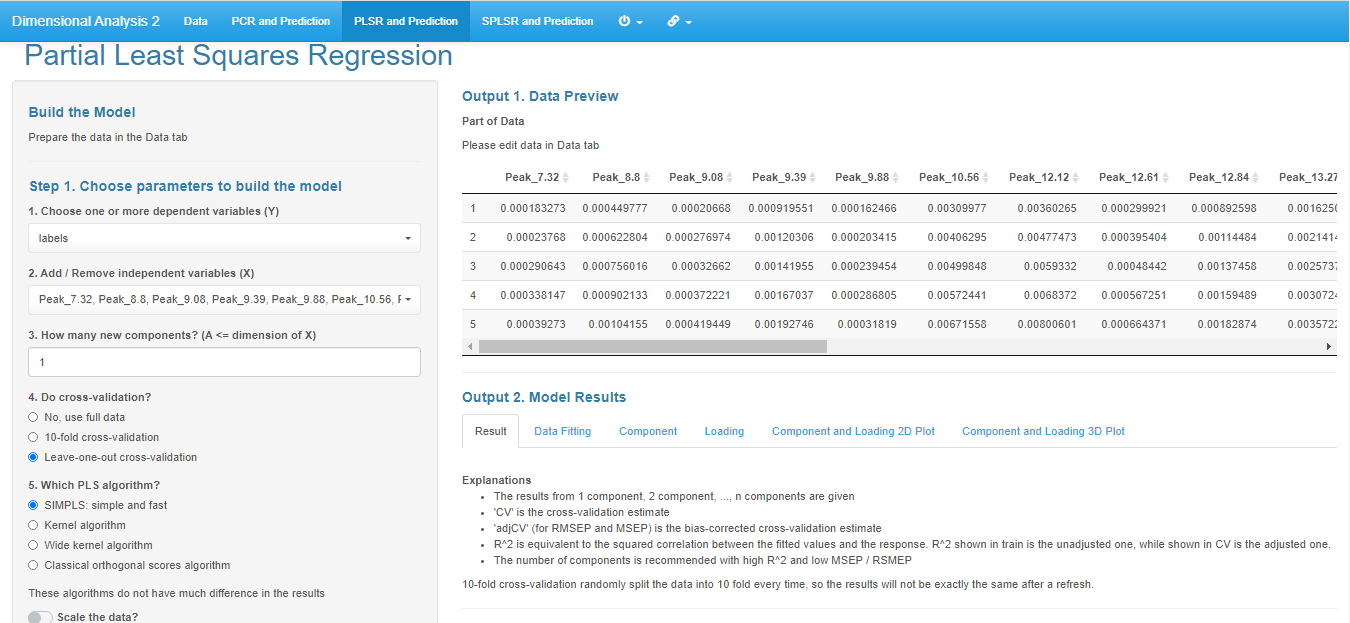


**Figure S3.** MEPHAS interface for the building of Partial Least Squares regression model.

**Principal component analysis**

The uploaded .CSV file was a matrix including the names of peaks in the first raw and the labels in the second column. The first column included a serial number starting from 1. The appropriate separator (i.e. comma, tab, semicolon) was selected in order to read the file correctly (**Figures S4 and S5**). Then we press the button “Go to build PCA Model”. We choose the independent variables (X) and the number of components. Components, scores plot and loadings are presented in “Output 2. Model Results” of Principal Component Analysis interface.

**Figure S4.** Part of the table uploaded to MEPHAS for the principal component analysis.


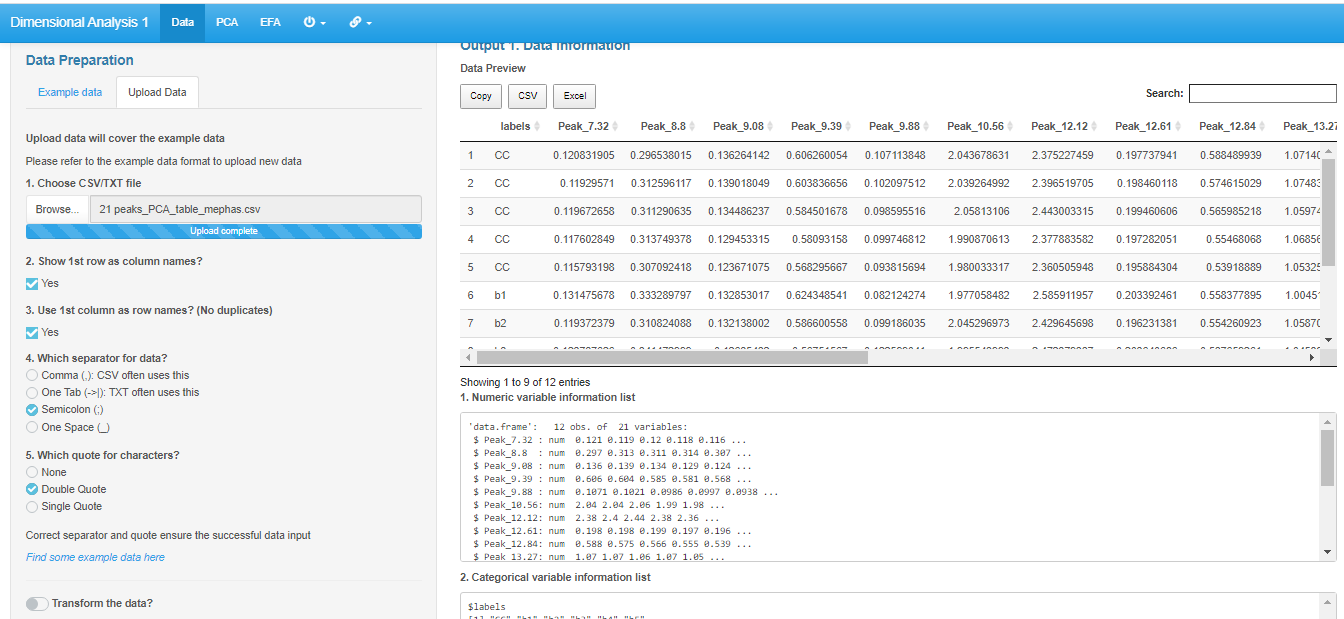


**Figure S5**. MEPHAS interface of the data preparation for the Principal Component Analysis.

1. **Past**

The uploaded .CSV file was a matrix including the names of peaks in the first raw and the labels in the first column (**Figure S6**). We check the boxes “Row attributes” and “Column attributes” in order to define the names of the variables and the samples. We uncheck the boxes before we proceed to the analysis. We perform Principal component analysis from the Multivariate menu.


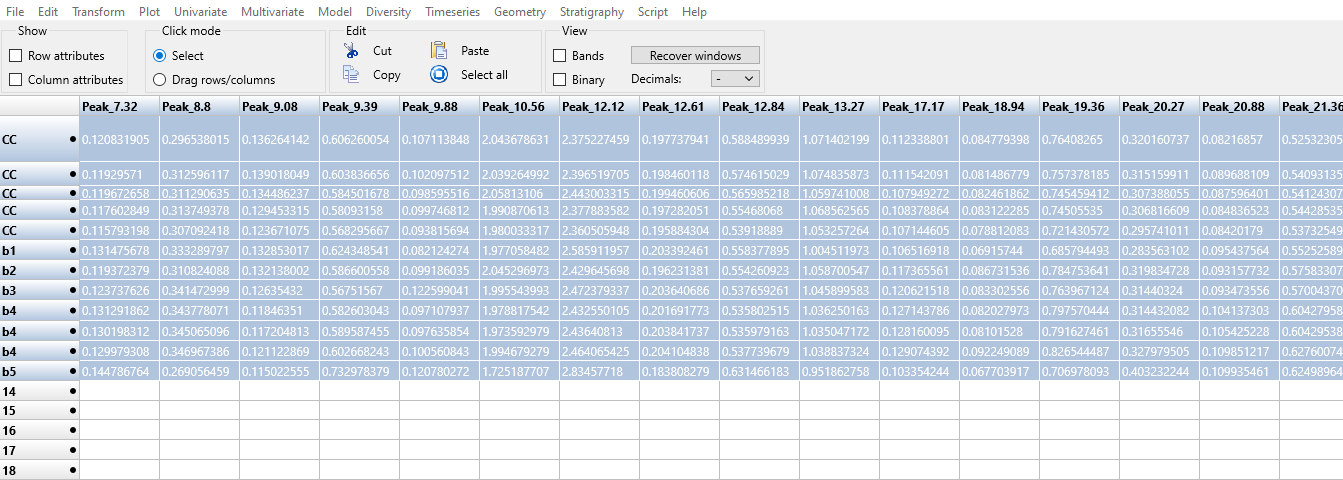


**Figure S6**. Imported data to the PAST software.

1. **Similarity tests**

The table that was imported to RStudio as .CSV file included the ratios of the peaks at rows. The ratios of the reference sample were located at the second row. The peak and samples names were included at the first row and the first column, respectively (**Figure S7**).

**Figure S7.** Part of the table imported to RStudio in order to perform the similarities tests using the “proxy” package.

> library(“proxy”)

> ratios_table <- read_delim("C:/Temp/ peak_ratios_table.csv", ";", escape_double = FALSE, trim_ws = TRUE)

> data <-ratios_table [,-1]

> samples <-ratios_table [,1]

> eJaccard_results <-as.matrix(as.matrix(simil(data, method = "eJaccard"))[1,])

> write.csv(eJaccard_results, file=" eJaccard_results.csv")

1. **Heatmaps and SSIM**

> library(stats)

> library(readr)

> table_heatmap_1 <- read_delim("C:/Temp/ table_heatmap_1.csv", ";", escape_double = FALSE, trim_ws = TRUE)

> table_heatmap_2 <- read_delim("C:/Temp/ table_heatmap_2.csv", ";", escape_double = FALSE, trim_ws = TRUE)

> heatmap_1 <-as.matrix(table_heatmap_1)

> heatmap_2 <-as.matrix(table_heatmap_2)

> library("RColorBrewer")

> coul <- colorRampPalette(brewer.pal(11, "RdYlBu"))(25)

> heatmap.1 <- heatmap(heatmap_1, Rowv = NA, Colv = NA, scale="none", col = coul)

> heatmap.2 <- heatmap(heatmap_2, Rowv = NA, Colv = NA, scale="none", col = coul)

> library("SPUTNIK")

> SSIM(heatmap.1, heatmap.2, numBreaks = 256)
