# Supplementary material for: A Novel Validated Injectable Colistimethate Sodium Analysis Combining Advanced Chemometrics and Design of Experiments
Source: Molecules. 2021 Mar 11;26(6):1546. doi: 10.3390/molecules26061546 (PMC8000333; doi:10.3390/molecules26061546)
Supplement: Supplementary file 1 [file molecules-26-01546-s001.zip › molecules-1135428-supplementary-final/Supplementary aó.docx]

**Supplementary B**


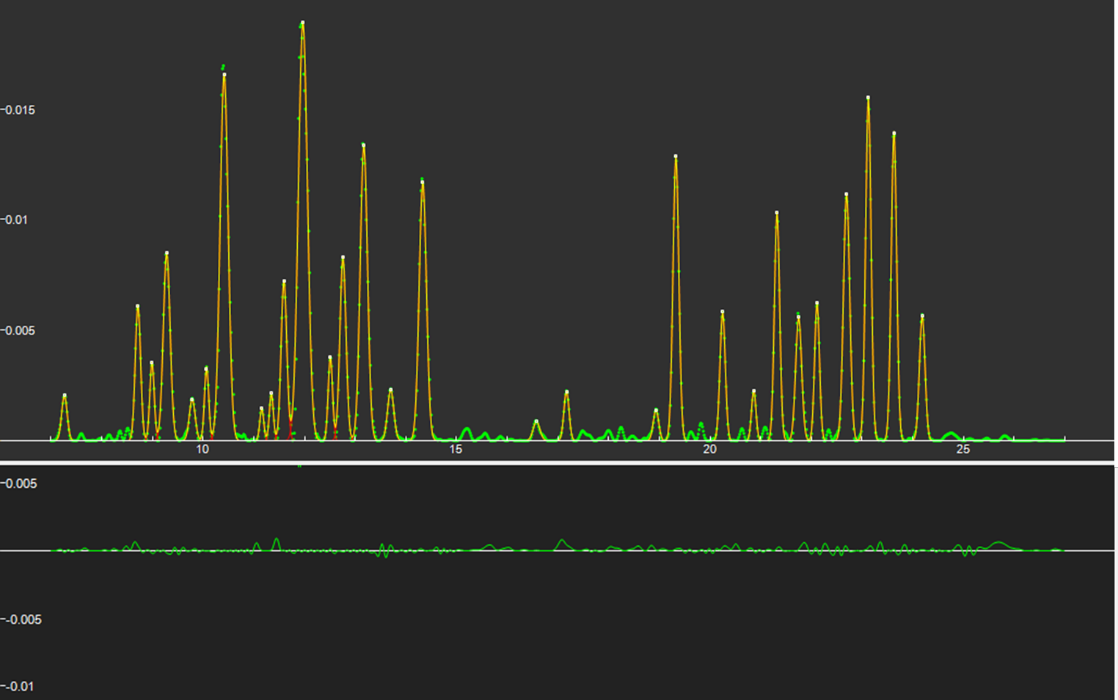


**Figure S1.** Peak fitting in the UPLC-UV chromatogram of 100 μg mL^-1^ CMS, employing the Fityk (v. 1.3.1) software. The residual plot above the chromatogram indicates the good fitting.


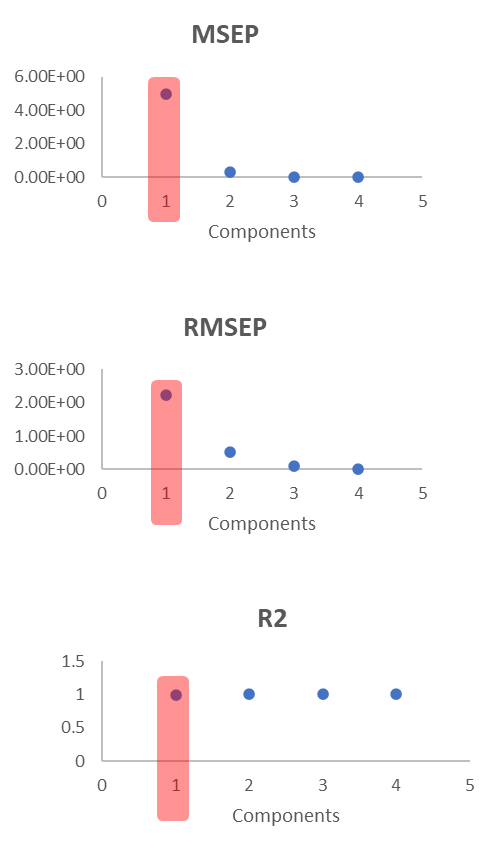


**Figure S 2.** The values of Mean square error of prediction (MSEP), Root mean square error of prediction (RMSEP) and R2 by using 1 – 4 components for PLSr model construction. It is observed that one component can describe fully the model.

**Table S1.** Interpolated values (mg mL^-1^) of the CMS components in the tested batches, as they were calculated by the linear regression models developed in MEPHAS. The upper and lower limits of the interpolated values at the 95% confidence level are also presented.

| **Batches** | | | | | | | | | | | | | | |
| --- | --- | --- | --- | --- | --- | --- | --- | --- | --- | --- | --- | --- | --- | --- |
| **Peaks** | **b1** | Upper Limit | **b2** | Upper Limit | **b3** | Upper Limit | **b4a** | Upper Limit | **b4b** | Upper Limit | **b4c** | Upper Limit | **b5** | Upper Limit |
|  |  | Lower Limit |  | Lower Limit |  | Lower Limit |  | Lower Limit |  | Lower Limit |  | Lower Limit |  | Lower Limit |
| **peak_7.28** | **172.37** | 174.45 | **160.18** | 162.15 | **177.90** | 180.07 | **197.40** | 200.10 | **203.45** | 206.36 | **183.76** | 186.07 | **215.34** | 218.69 |
|  |  | 170.35 |  | 158.21 |  | 175.80 |  | 194.85 |  | 200.73 |  | 181.56 |  | 212.22 |
| **peak_8.73** | **166.43** | 169.85 | **159.52** | 162.86 | **186.13** | 190.22 | **195.02** | 199.58 | **203.04** | 208.07 | **185.78** | 189.86 | **151.79** | 155.14 |
|  |  | 163.09 |  | 156.18 |  | 182.37 |  | 190.91 |  | 198.54 |  | 182.03 |  | 148.33 |
| **peak_9.02** | **155.89** | 163.19 | **159.66** | 167.04 | **163.45** | 170.98 | **159.12** | 166.49 | **164.16** | 171.72 | **152.44** | 159.72 | **151.37** | 158.66 |
|  |  | 148.34 |  | 152.25 |  | 156.14 |  | 151.70 |  | 156.86 |  | 144.70 |  | 143.56 |
| **peak_9.3** | **165.36** | 168.19 | **159.00** | 161.78 | **164.58** | 167.40 | **176.96** | 180.04 | **186.60** | 190.00 | **172.23** | 175.18 | **222.71** | - |
|  |  | 162.58 |  | 156.21 |  | 161.80 |  | 174.04 |  | 183.42 |  | 169.38 |  | - |
| **peak_9.8** | **123.53** | 128.94 | **158.68** | 163.06 | **216.73** |  | **174.85** | 179.66 | **183.56** | 188.84 | **170.11** | 174.73 | **219.82** | - |
|  |  | 117.34 |  | 154.27 |  | 209.98 |  | 170.35 |  | 178.79 |  | 165.71 |  | 212.84 |
| **peak_10.43** | **151.30** | 154.88 | **161.01** | 164.58 | **167.91** | 171.59 | **173.71** | 177.56 | **180.11** | 184.20 | **164.75** | 168.37 | **148.58** | 152.19 |
|  |  | 147.60 |  | 157.45 |  | 164.34 |  | 170.06 |  | 176.31 |  | 161.20 |  | 144.81 |
| **peak_11.99** | **167.38** | 170.75 | **161.37** | 164.66 | **175.33** | 178.92 | **179.80** | 183.54 | **187.03** | 191.09 | **171.63** | 175.10 | **206.85** | 212.03 |
|  |  | 164.09 |  | 158.09 |  | 171.93 |  | 176.29 |  | 183.29 |  | 168.29 |  | 202.23 |
| **peak_12.53** | **159.21** | 161.07 | **157.64** | 159.50 | **174.67** | 176.67 | **180.31** | 182.41 | **189.25** | 191.57 | **171.96** | 173.91 | **162.05** | 163.91 |
|  |  | 157.35 |  | 155.78 |  | 172.73 |  | 178.29 |  | 187.05 |  | 170.05 |  | 160.19 |
| **peak_12.76** | **153.46** | 155.25 | **156.65** | 158.43 | **162.76** | 164.56 | **169.68** | 171.53 | **176.89** | 178.84 | **159.61** | 161.40 | **200.00** | 202.53 |
|  |  | 151.64 |  | 154.85 |  | 160.98 |  | 167.86 |  | 174.99 |  | 157.82 |  | 197.62 |
| **peak_13.18** | **145.82** | 147.84 | **157.86** | 159.79 | **166.52** | 168.49 | **171.98** | 174.02 | **178.42** | 180.57 | **162.42** | 164.37 | **155.72** | 157.66 |
|  |  | 143.75 |  | 155.91 |  | 164.57 |  | 169.99 |  | 176.34 |  | 160.49 |  | 153.76 |
| **peak_14.35** | **154.64** | 156.91 | **158.66** | 160.92 | **169.20** | 171.54 | **176.24** | 178.72 | **182.93** | 185.57 | **166.23** | 168.54 | **173.91** | 176.33 |
|  |  | 152.34 |  | 156.39 |  | 166.90 |  | 173.86 |  | 180.42 |  | 163.96 |  | 171.56 |
| **peak_17.18** | **150.79** | 153.89 | **171.50** | 174.76 | **188.85** | 192.73 | **208.18** | 213.11 | **218.25** | - | **198.88** | 203.27 | **165.47** | 168.61 |
|  |  | 147.59 |  | 168.37 |  | 185.27 |  | 203.77 |  | 213.31 |  | 194.90 |  | 162.39 |
| **peak_18.95** | **129.14** | 133.80 | **168.93** | 173.09 | **173.40** | 177.71 | **178.19** | 182.69 | **182.98** | 187.73 | **189.46** | 194.58 | **143.35** | 147.48 |
|  |  | 123.93 |  | 164.94 |  | 169.34 |  | 174.01 |  | 178.65 |  | 184.86 |  | 138.91 |
| **peak_19.33** | **141.66** | 143.62 | **168.03** | 169.91 | **174.95** | 176.93 | **191.18** | 193.52 | **197.33** | 199.84 | **186.53** | 188.74 | **166.00** | 167.86 |
|  |  | 139.63 |  | 166.18 |  | 173.04 |  | 188.96 |  | 194.95 |  | 184.41 |  | 164.16 |
| **peak_20.27** | **141.25** | 143.54 | **165.80** | 167.97 | **174.65** | 176.95 | **182.64** | 185.12 | **191.58** | 194.33 | **179.32** | 181.72 | **234.93** | - |
|  |  | 138.86 |  | 163.65 |  | 172.43 |  | 180.27 |  | 189.00 |  | 177.02 |  | - |
| **peak_20.87** | **172.14** | 180.78 | **172.45** | 181.12 | **184.84** | 194.88 | **214.98** | - | **226.10** | - | **213.74** | - | **224.00** | - |
|  |  | 164.34 |  | 164.65 |  | 176.52 |  | 203.83 |  |  |  | 202.73 |  |  |
| **peak_21.33** | **158.55** | 160.51 | **169.28** | 171.30 | **178.59** | 180.77 | **196.83** | 199.50 | **204.21** | 207.14 | **192.78** | 195.32 | **200.75** | 203.56 |
|  |  | 156.59 |  | 167.29 |  | 176.49 |  | 194.31 |  | 201.47 |  | 190.37 |  | 198.12 |
| **peak_22.75** | **144.83** | 149.00 | **166.24** | 170.40 | **174.96** | 179.40 | **191.92** | 197.30 | **197.88** | 203.68 | **185.96** | 190.96 | **194.90** | 200.48 |
|  |  | 140.38 |  | 162.19 |  | 170.79 |  | 187.13 |  | 192.78 |  | 181.43 |  | 189.95 |
| **peak_22.12** | **140.39** | 143.68 | **166.33** | 169.47 | **166.10** | 169.25 | **179.57** | 183.06 | **186.70** | 190.48 | **175.73** | 179.10 | **239.08** |  |
|  |  | 136.90 |  | 163.25 |  | 163.02 |  | 176.28 |  | 183.20 |  | 172.53 |  |  |
| **peak_22.7** | **147.71** | 149.54 | **166.63** | 168.44 | **170.62** | 172.47 | **186.55** | 188.69 | **194.49** | 196.84 | **183.81** | 185.88 | **211.31** | 214.18 |
|  |  | 145.84 |  | 164.85 |  | 168.81 |  | 184.50 |  | 192.26 |  | 181.81 |  | 208.61 |
| **peak_23.13** | **133.52** | 135.87 | **165.97** | 168.05 | **173.85** | 176.04 | **183.17** | 185.56 | **188.17** | 190.70 | **178.13** | 180.40 | **134.49** | 136.82 |
|  |  | 131.04 |  | 163.91 |  | 171.73 |  | 180.89 |  | 185.77 |  | 175.94 |  | 132.03 |
| **peak_23.65** | **130.46** | 133.82 | **163.74** | 166.63 | **168.55** | 171.51 | **180.30** | 183.56 | **185.57** | 189.03 | **173.56** | 176.62 | **137.05** | 140.20 |
|  |  | 126.84 |  | 160.88 |  | 165.67 |  | 177.22 |  | 182.34 |  | 170.62 |  | 133.69 |
| **peak_24.2** | **166.95** | 174.05 | **176.09** | 183.82 | **179.92** | 187.99 | **198.30** | 208.54 | **206.27** | 217.64 | **193.16** | 202.72 | **234.58** |  |
|  |  | 160.21 |  | 169.20 |  | 172.88 |  | 190.05 |  | 197.30 |  | 185.32 |  |  |

.

**Table S2.** Eigenvalues, % variances, Eig 2.5% and Eig 97.5% of the principal componets after 7-fold bootstrapping, as they were calculated from the PAST software during the Principal Component Analysis.

| **PC** | **Eigenvalue** | **% variance** | **Eig 2.5%** | **Eig 97.5%** |
| --- | --- | --- | --- | --- |
| 1 | 0.035942 | 79.772 | 56.641 | 83.07 |
| 2 | 0.005786 | 12.842 | 3.4428 | 24.065 |
| 3 | 0.001707 | 3.7885 | 2.97E-28 | 3.4648 |
| 4 | 0.00113 | 2.5082 | 1.70E-28 | 4.4464 |
| 5 | 0.000249 | 0.55169 | 0.11774 | 1.3485 |
| 6 | 0.00017 | 0.37746 | 0 | 0.68779 |
| 7 | 4.30E-05 | 0.095444 | 0 | 0.32695 |
| 8 | 1.85E-05 | 0.041069 | 0 | 0.033566 |
| 9 | 5.14E-06 | 0.011403 | 0 | 5.24E-34 |
| 10 | 4.31E-06 | 0.009564 | 0 | 2.74E-33 |
| 11 | 8.79E-07 | 0.00195 | 0 | 0 |


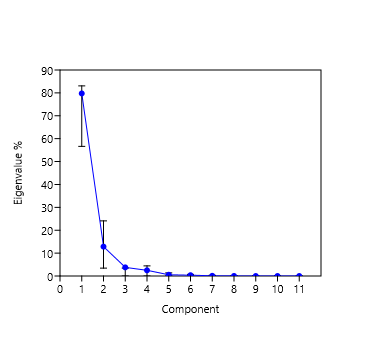


**Figure S3.** Scree plot as it is produced by PAST software.
